# Generalizable Compositional Features Influencing the Proteostatic Fates of Polar Low-Complexity Domains

**DOI:** 10.3390/ijms22168944

**Published:** 2021-08-19

**Authors:** Sean M. Cascarina, Joshua P. Kaplan, Mikaela R. Elder, Lindsey Brookbank, Eric D. Ross

**Affiliations:** Department of Biochemistry and Molecular Biology, Colorado State University, Fort Collins, CO 80525, USA; Sean.Cascarina@colostate.edu (S.M.C.); kaplanjp@usc.edu (J.P.K.); mikaela.elder@vanderbilt.edu (M.R.E.); lindseybrookbank@gmail.com (L.B.)

**Keywords:** proteostasis, protein aggregation, prion, protein degradation, amyloid, ubiquitin-proteasome system, protein misfolding, low-complexity domain, neurodegenerative disorder

## Abstract

Protein aggregation is associated with a growing list of human diseases. A substantial fraction of proteins in eukaryotic proteomes constitutes a proteostasis network—a collection of proteins that work together to maintain properly folded proteins. One of the overarching functions of the proteostasis network is the prevention or reversal of protein aggregation. How proteins aggregate in spite of the anti-aggregation activity of the proteostasis machinery is incompletely understood. Exposed hydrophobic patches can trigger degradation by the ubiquitin-proteasome system, a key branch of the proteostasis network. However, in a recent study, we found that model glycine (G)-rich or glutamine/asparagine (Q/N)-rich prion-like domains differ in their susceptibility to detection and degradation by this system. Here, we expand upon this work by examining whether the features controlling the degradation of our model prion-like domains generalize broadly to G-rich and Q/N-rich domains. Experimentally, native yeast G-rich domains in isolation are sensitive to the degradation-promoting effects of hydrophobic residues, whereas native Q/N-rich domains completely resist these effects and tend to aggregate instead. Bioinformatic analyses indicate that native G-rich domains from yeast and humans tend to avoid degradation-promoting features, suggesting that the proteostasis network may act as a form of selection at the molecular level that constrains the sequence space accessible to G-rich domains. However, the sensitivity or resistance of G-rich and Q/N-rich domains, respectively, was not always preserved in their native protein contexts, highlighting that proteins can evolve other sequence features to overcome the intrinsic sensitivity of some LCDs to degradation.

## 1. Introduction

Protein homeostasis, or “proteostasis”, encompasses the set of processes involved in the proper production and maintenance of cellular proteins. Proteostasis in eukaryotic cells is achieved through the coordinated action of three main branches—protein chaperones, the ubiquitin-proteasome system, and autophagy—which work together to achieve and maintain native protein folds, prevent protein aggregation, and degrade damaged or misfolded proteins (for review, see [1,2]).

Under native conditions, most proteins must remain soluble to carry out their normal function(s) in cells. While proteostasis systems are generally quite effective at producing and maintaining thousands or tens of thousands of proteins with diverse sequences and folds, misfolding and aggregation are frequently observed in a variety of organisms. Failure of the proteostasis machinery can be broadly attributed to four main causes (which are not mutually exclusive; [1]): (1) the capacity of the proteostasis machinery may be exceeded by widespread protein misfolding or by a single protein with extremely high aggregation propensity or aggregate persistence; (2) mutations in components of the proteostasis network can reduce the overall capacity of the network; (3) a systemic decline in proteostasis, often associated with age, can result in accumulation of damaged or misfolded proteins; or (4) inherent sequence or structural features may favor evasion of the proteostasis defenses.

Although cells devote an enormous amount of energy and resources to maintaining proteostasis, failures of the proteostasis machinery are not uncommon. Protein misfolding and aggregation are associated with a variety of pathologies, including diabetes, neurodegeneration, myopathy, and some cancers [3]. Since protein aggregation is often deleterious, many proteostasis factors specifically recognize aggregation-prone protein features and either re-fold or degrade the protein [4,5,6,7,8,9,10,11,12]. Therefore, protein aggregates observed in vivo have likely formed and persisted in the presence of proteostasis factors whose primary role is to prevent or clear misfolded proteins. This suggests that the current understanding of protein aggregation may be influenced by a subtle form of observation bias: proteins capable of aggregating in vivo may do so not only because they possess aggregation-prone features (which have classically been the focus of protein aggregation studies) but also because these features may be inadequately recognized by the proteostasis machinery. Understanding the molecular features influencing the proteostatic regulation of aggregation-prone proteins may be as important as understanding the aggregation-prone features themselves.

Prion proteins constitute a unique class of aggregation-prone proteins, defined by the ability to form infectious, self-propagating aggregates [13]. The domains responsible for prion activity are typically low-complexity domains (LCDs), which are broadly defined as regions with unusually biased amino acid compositions [14]. Specifically, yeast prion domains (PrDs) are a subcategory of LCDs enriched in Q/N residues, with secondary biases for G, S, and/or Y [14,15,16,17]. These features have been incorporated into multiple prion prediction algorithms [16,17,18,19,20,21,22] and have aided in the identification of PrDs and prion-like domains (PrLDs) across a variety of organisms [16,17,22,23,24,25,26,27,28,29,30].

In a recent study, we utilized model G-rich PrLDs from hnRNPA1 and hnRNPA2, as well as a classical PrD from the yeast prion protein, Sup35, to delineate sequence features that control the proteostatic fates of aggregation-prone LCDs [31]. Specifically, we substituted the G-rich LCDs from hnRNPA1 and hnRNPA2 in place of the Q/N-rich nucleation domain from Sup35. We then mutated these fusion proteins and used Sup35 activity to monitor protein stability and aggregation. While sequence features that promote protein aggregation were generally correlated with detection by the proteostasis machinery, this relationship could be uncoupled in multiple ways. First, we found that insertion of non-aromatic hydrophobic residues into aggregation-prone G-rich PrLDs caused the proteins to be efficiently recognized and degraded by the ubiquitin-proteasome system, whereas identical sequences within a native yeast Q/N-rich PrD from Sup35 increased its aggregation propensity without affecting its rate of clearance by the ubiquitin-proteasome system. Progressive Q/N→G substitution surrounding inserted hydrophobic residues led to a loss of degradation resistance, suggesting that Q/N-rich regions can mask potent degradation-promoting peptides (“degrons”) from the ubiquitin-proteasome machinery. Second, we found that aromatic residues could increase the aggregation propensity of the G-rich PrLDs without substantially affecting their degradation rate.

While our model G-rich and Q/N-rich LCDs facilitated an in-depth exploration of the molecular features governing their aggregation or proteostatic regulation, it is unclear whether these heuristics only apply to the model PrLDs. Here, we leverage a combination of bioinformatic and experimental techniques to explore whether the molecular rules governing the aggregation or degradation of our model PrLDs generalize to other polar LCDs. Our analyses of the yeast and human proteomes suggest that constraints imposed via the proteostasis machinery may help to determine compositional biases among different classes of LCDs. Additionally, we find that native G-rich LCDs are broadly susceptible to the degradation-promoting effect of hydrophobic residues, whereas native Q/N-rich LCDs consistently mask this effect. However, the proteostatic regulation of these G-rich and Q/N-rich LCDs did not always confer similar degradation susceptibility or resistance in the context of their native proteins, indicating that additional features may modulate this behavior. Finally, we also observe differential sensitivity to hydrophobic degrons for S-rich and T-rich LCDs. These results illuminate the interplay between compositional features and proteostatic fates for polar LCDs in eukaryotes.

## 2. Results

### 2.1. Sequence Preferences within Native G-Rich and Q/N-Rich LCDs Suggest Proteostatic Constraints on Allowable Sequence Space

**Figure 1:**
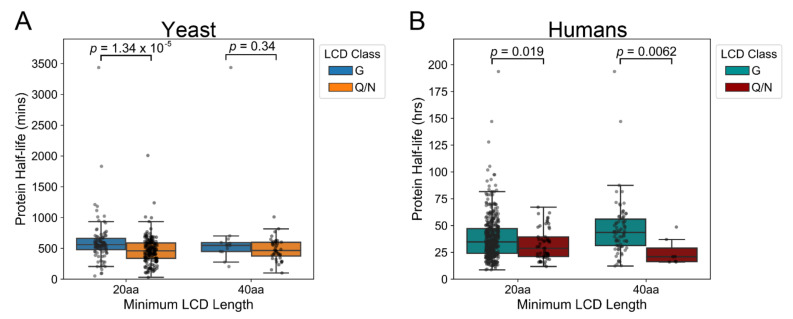
Native yeast and human proteins with G-rich LCDs have longer half-lives than proteins with Q/N-rich LCDs. Native yeast (**A**) and human (**B**) proteins containing G-rich or Q/N-rich LCDs were identified with the LCD-Composer algorithm using a short (20 aa) and long (40 aa) window size. G-rich LCDs were defined as regions with ≥35% G (the approximate G content of the hnRNP PrLDs), whereas Q/N-rich LCDs were defined as regions with ≥50% Q/N (the approximate Q/N content of the Sup35 ND). Half-life values were derived from [37,38].

Despite the sensitivity of our model G-rich LCDs to degradation, a recent analysis of LCD-containing proteins showed that native yeast proteins with G-rich LCDs actually tend to have longer half-lives compared to non-G-rich proteins [32]. Additionally, despite the apparent resistance of Q/N-rich LCDs to degradation, native proteins with Q/N-rich LCDs tend to have shorter half-lives compared to non-Q/N-rich proteins [32]. However, the degree of G or Q/N enrichment in our model LCDs was not equivalent, and the relative difference between G-rich and Q/N-rich LCDs is not readily interpretable from these analyses. In order to make a direct comparison of G-rich and Q/N-rich proteins, we exhaustively scanned the yeast proteome for domains resembling our original model substrates, the hnRNP PrLDs [33,34] and the Sup35 nucleation domain (ND; a critical portion of the larger PrD [35]), with respect to both minimum size and minimum G or Q/N composition (see Materials and Methods). Indeed, proteins with G-rich LCDs tend to have longer half-lives than proteins with Q/N-rich LCDs in both yeast and humans when these two groups are compared directly (Figure 1 and Tables S1 and S2; see Materials and Methods for definition of G-rich and Q/N-rich LCDs; see Supplementary Materials). This observation and our previous data [31,36] imply that proteins with native Q/N-rich LCDs are not inherently more resistant to proteolysis than proteins with G-rich LCDs in vivo. Therefore, we hypothesized that secondary sequence features within native G-rich and Q/N-rich LCDs (i.e., non-G and non-Q/N residues, respectively) might influence the half-lives of their parent proteins.

To explore this possibility, we first sought to more accurately define the compositional features that promote protein degradation within our model LCDs. In our previous study [31], we utilized versions of Sup35 in which a portion of the Sup35 prion domain was replaced with the PrLDs from hnRNPA1 and hnRNPA2. To examine how compositional changes affect protein turnover and prion formation, we replaced an 8-amino acid segment within the PrLDs with a library of random sequences. We found that many members of the library showed low levels of Sup35 activity and that this low activity was consistently due to high rates of protein degradation for Sup35. While these initial studies were sufficient to identify general features correlated with protein degradation, the sample sizes were insufficient to provide accurate degradation propensity scores for individual amino acids. Therefore, using our previous mutagenesis and phenotypic screening pipeline [31], we expanded the sequence libraries for the hnRNPA1 and hnRNPA2 PrLDs (Table S3). For nearly all amino acids in both libraries, expansion of the dataset did not dramatically influence degradation propensity scores. However, *p*-values corresponding to the degradation propensity scores tended to decrease for amino acids having the greatest effect on degradation, suggesting improved confidence for these residues. Additionally, the correlation (R^2^) between the hnRNPA1 and hnRNPA2 library scores is markedly higher than observed previously (Figure S1; [31]), indicating that the same features control the degradation of both hnRNP G-rich LCDs. Therefore, to generate a unified degradation propensity scale and account for possible context-specific effects unique to either hnRNP, we combined the amino acid frequencies from the two mutagenesis libraries and calculated a single degradation propensity for each amino acid (Table S3).

**Figure 2:**
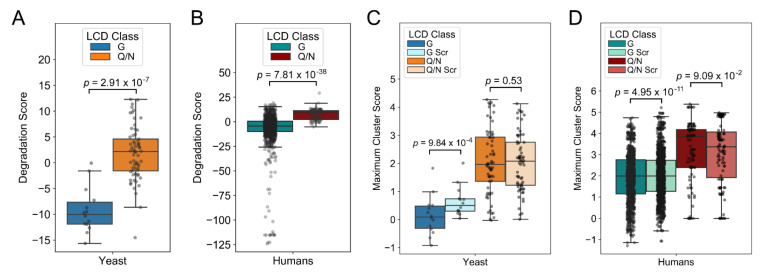
Comparison of cumulative degradation scores and maximum cluster scores G-rich and Q/N-rich LCDs. Native G-rich LCDs have significantly lower cumulative degradation scores (two-tailed Mann–Whitney *U* test) than Q/N-rich LCDs in yeast (**A**) and humans (**B**). (**C**) Scrambled yeast G-rich LCDs have significantly higher maximum cluster scores (8 aa cluster size) than their native counterparts (two-sided, paired *t*-test; see Materials and Methods). (**D**) Scrambled human G-rich LCDs have similar or slightly higher maximum cluster scores than native G-rich LCDs, whereas scrambled Q/N-rich LCDs have similar or slightly lower maximum cluster scores relative to their native counterparts. For all panels, native LCDs were identified using LCD-Composer with a 40 aa window size.

Initial bioinformatic analyses suggested that G-rich LCDs tended to have fewer strongly degradation-promoting amino acids than Q/N-rich LCDs [36]. However, this preliminary analysis does not account for the cumulative degradation propensity of all 20 amino acids in the context of G-rich and Q/N-rich LCDs. When scored with our improved degradation propensity scores, secondary features within native yeast and human G-rich LCDs result in significantly lower overall scores when compared to Q/N-rich LCDs (excluding G and Q/N, respectively), indicating that the global sequence composition of native yeast G-rich LCDs may naturally prevent susceptibility to high turnover rates (Figure 2A,B).

Degrons are often short linear motifs [39,40,41] with high degradation propensity, so clustering of degradation-promoting amino acids within G-rich and Q/N-rich LCDs may influence their potency. Furthermore, Q and N both exhibit low degradation propensity scores [31], so hydrophobic residues may be rendered incapable of inducing degradation when flanked by Q/N residues within Q/N-rich LCDs. To examine whether G-rich or Q/N-rich LCDs avoid clustering of residues with high degradation propensity, we repeatedly scrambled the original sequence of each domain and calculated the average maximum 8 aa score, excluding the primary amino acid(s) of interest (either G or Q/N; see Materials and Methods). Scrambled yeast G-rich LCDs have significantly higher cluster scores than native yeast G-rich LCDs (Figure 2C), suggesting that G-rich LCDs indeed selectively avoid clustering of degradation-promoting amino acids. However, little difference is observed between scrambled and native human G-rich LCDs (despite a statistically significant *p*-value; Figure 2D). Conversely, scrambled Q/N-rich LCDs have similar or slightly lower cluster scores than native Q/N-rich LCDs in yeast and humans (Figure 2C,D). Similar trends are observed with a shorter 4 aa cluster size (Figure S2). Collectively, these results suggest that selective pressures limit the overall degradation propensity scores as well as the clustering of degradation-inducing amino acids within eukaryotic G-rich LCDs, whereas Q/N-rich LCDs exhibit no discernible susceptibility to these pressures.

### 2.2. Generalizable Regulatory Principles Govern Aggregation or Degradation of Native Yeast G-Rich and Q/N-Rich LCDs

Our bioinformatic analyses suggest that the principles governing degradation or stability observed for our model G-rich and Q/N-rich LCDs, respectively, may apply broadly to these classes of LCDs. However, our degradation propensity scores were founded upon an assay that was developed using model G-rich human PrLDs, which were subsequently compared to yeast Q/N-rich domains. A major remaining question is whether these degradation heuristics would apply in the same manner to native yeast G-rich domains, and whether additional native Q/N-rich domains would similarly suppress hydrophobic degrons.

In order to examine this directly, we identified native yeast LCDs of comparable length and with G or Q/N content near to or exceeding those of the hnRNP PrLDs or Sup35 ND, respectively, using the LCD-Composer algorithm ([14]; see Materials and Methods). G-rich LCDs of this size and minimum G composition are relatively rare within the yeast proteome, whereas comparable Q/N-rich LCDs are more abundant [16,22,36]. We initially chose four native yeast domains to test: two G-rich LCDs from Ynl208W and Npl3, and two Q/N-rich LCDs from two other yeast prion proteins Rnq1 and Ure2. Coincidentally, Ynl208W has a moderately Q/N-rich domain at its N-terminus (entirely distinct from the G-rich domain at its C-terminus), allowing us to test both a G-rich and Q/N-rich domain from the same protein.

The Sup35 fusion system is widely used to assess prion aggregation (and, more recently, degradation [31]) of candidate PrLDs [17,25,27,33,42,43,44,45]. Sup35 is involved in the release of nascent polypeptides from the ribosome during translation termination [46,47]. Fully functional Sup35 in an *ade2-1* genetic background leads to the expression of a non-functional, truncated form of the Ade2 protein required for adenine biosynthesis. These strains are unable to grow on media lacking adenine (*ade*^−^ phenotype) and appear red on media with limited adenine. Compromised Sup35 function, whether by prion aggregation or enhanced protein turnover, increases read-through of the premature stop codon in the *ade2-1* transcript, resulting in cells that are able to grow on media lacking adenine (*ADE*^+^ phenotype) and appear white on media with limited adenine. Importantly, the prion domain of Sup35 is spatially and functionally distinct from the domain responsible for chain release during translation termination [48]. Therefore, the Sup35 translation termination activity can be used as a reporter for candidate PrLD-dependent degradation or aggregation of Sup35.

**Figure 3:**
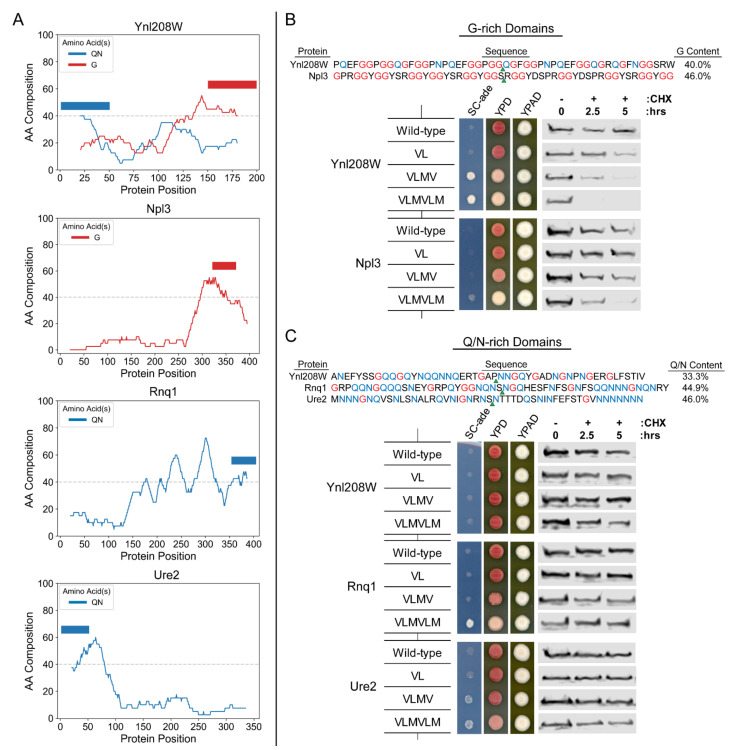
Sequence features controlling the proteostatic regulation of model PrLDs generalize to native G-rich and Q/N-rich LCDs. (**A**) Composition plots for selected G-rich and Q/N-rich proteins were generated using CompositionPlotter (https://github.com/RossLabCSU/IJMS_2021, accessed on 19 April 2021). Solid bars above the line plots indicate the G-rich and Q/N-rich domains selected for experimental testing. The amino acid sequences of native G-rich (**B**; top) and Q/N-rich (**C**; top) domains, as well as G or Q/N composition and hydrophobic insertion sites (green arrowheads), are indicated. (**B**) Progressive insertion of hydrophobic residues within native G-rich LCDs leads to the emergence of the *ADE*^+^ phenotype (left) and a corresponding increase in degradation rate (right). (**C**) Insertion of hydrophobic residues within two Q/N-rich yeast prion domains leads to the appearance of sectoring (Rnq1), a classical indication of spontaneous prion formation), or to a weak *ADE*^+^ phenotype in a subset of cells (Ure2). Identical insertions in the Ynl208W Q/N-rich domain are consistently associated with an *ade*^−^ phenotype, suggesting no detectable increase in degradation or spontaneous prion formation. Critically, insertion of hydrophobic residues in all Q/N-rich LCDs tested did not detectably enhance their degradation rates within 5 h after addition of CHX.

We took advantage of the Sup35 fusion system by replacing the Sup35 ND with each of the native G-rich or Q/N-rich LCDs and subsequently inserted two, four, and six hydrophobic residues ~25 residues from the N-terminus within each domain (Figure 3). Chimeric proteins with the wild-type (WT) G-rich LCDs from Ynl208W and Npl3 are relatively stable and the cells are correspondingly *ade*^−^ (Figure 3B and Figure S3), indicating that these domains are not inherently degradation-inducing. However, insertion of four or six hydrophobic residues in the G-rich domain from Ynl208W results in the appearance of a robust *ADE*^+^ phenotype and a concomitant increase in degradation rate. Insertion of six hydrophobic residues in the G-rich domain from Npl3 also results in the appearance of the *ADE*^+^ phenotype and an increase in degradation rate. By contrast, all of the Q/N-rich LCDs are resistant to degradation with up to six hydrophobic residues (the maximum number tested; Figure 3C and Figure S3). Furthermore, insertion of hydrophobic residues into the prion domains of the two known yeast prion proteins, Rnq1 and Ure2, resulted in a step-wise increase in the frequency of spontaneous prion formation (indicated by sectoring on YPD and growth on SC-ade), as observed for the native Sup35 ND [31]. No sectoring or *ADE*^+^ growth was observed upon insertion of hydrophobic residues into the Q/N-rich domain from Ynl208W, consistent with its lower predicted prion propensity despite moderate Q/N-enrichment (Figure S4): however, rare prion formation can be detected upon insertion of six hydrophobic residues when cells are plated at higher densities, as demonstrated in a later section.

Serial dilutions on selective media (SC-ade) provide a more sensitive indication of the proportion of ADE^+^ cells and their rate of growth, and can often distinguish degradation-related ADE^+^ growth from prion-related ADE^+^ growth. Typically, enhanced degradation of Sup35 fusions appears, at least phenotypically, to be relatively uniform among a population of cells and results in robust growth on SC-ade for the entire population of cells plated [31]. By contrast, spontaneous prion formation is a stochastic event and is generally only observed in a subset of cells among a population. Additionally, prions formed by Sup35 are cured with 4mM guanidine hydrochloride (GuanHCl) [49], which can distinguish prion-based and degradation-based ADE^+^ phenotypes.

**Figure 4:**
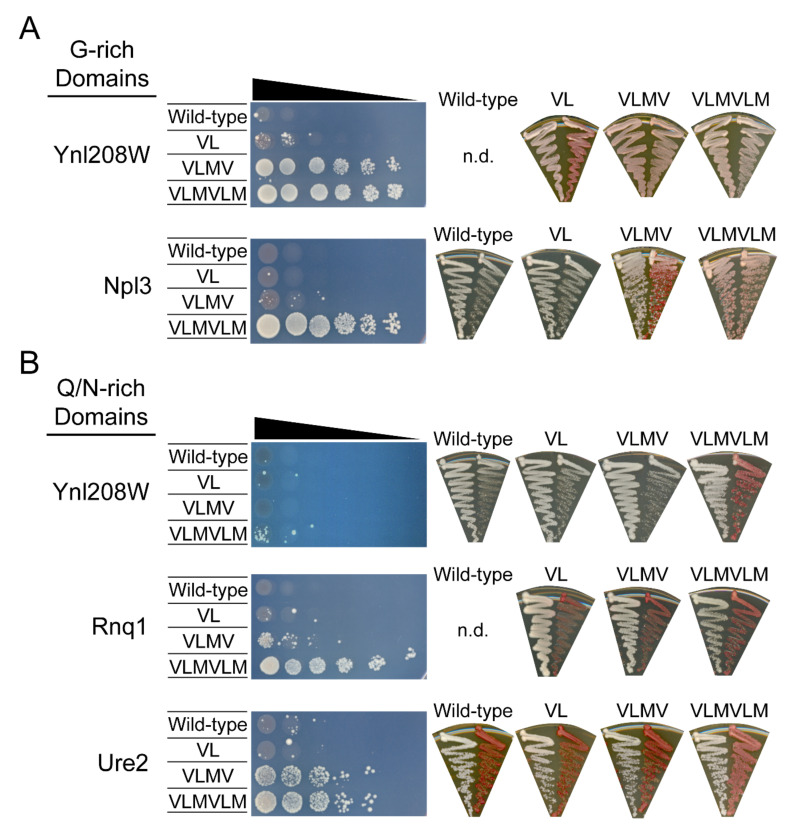
G-rich LCDs are associated with prion aggregation or degradation phenotypes, whereas Q/N-rich LCDs are exclusively associated with prion aggregation phenotypes. Strains expressing native G-rich LCDs (**A**) or native Q/N-rich LCDs (**B**) fused to Sup35 were plated in 10-fold serial dilution on SC-ade. (**A**) Insertion of hydrophobic residues within native G-rich LCDs leads to sharp increases in growth on SC-ade (left) at +4 hydrophobic residues (Ynl208W) and +6 hydrophobic residues (Npl3), and this *ADE*^+^ phenotype was consistently incurable by GuanHCl (right). However, insertion of two hydrophobic residues (Ynl208W) or four hydrophobic residues (Npl3), just below the threshold for the characteristic degradation-associated phenotype, led to infrequent growth on SC-ade that was sometimes cured by GuanHCl, indicative of prion formation. (**B**) Insertion of hydrophobic residues led to progressive increases in growth on SC-ade for the Rnq1 and Ure2 Q/N-rich LCDs but not for the Ynl208W Q/N-rich domain (for which growth on SC-ade was only observed upon insertion of six hydrophobic residues). The *ADE*^+^ phenotype for Rnq1 and Ure2 isolates was consistently curable, indicating prion formation as the predominant cause of growth on SC-ade. Rare colonies for Ynl208W with six hydrophobic residues were frequently curable. No data (“n.d.”) were obtained for the Ynl208W G-rich wild-type domain (colonies were inviable on GuanHCl) and Rnq1 wild-type domain (no colonies were viable on SC-ade).

Strains expressing each of the five native G-rich or Q/N-rich LCDs substituted in place of the Sup35 ND were plated in 10-fold serial dilution on SC-ade (Figure 4). The strain expressing the WT G-rich domain from Ynl208W in place of the Sup35 ND exhibits almost no growth on SC-ade (Figure 4A, left). Insertion of two hydrophobic residues results in at least ten-fold greater growth on SC-ade. For a proportion of ADE^+^ isolates, treatment with GuanHCl resulted in a transition to the *ade*^−^ phenotype (Figure 4A, right), indicating that insertion of two hydrophobic residues within the Ynl208W G-rich LCD could induce spontaneous prion formation. Strikingly, insertion of four or six hydrophobic residues resulted in an abrupt ~10,000-fold increase in the frequency of ADE^+^ colony growth. However, the ADE^+^ phenotype was maintained after GuanHCl treatment for all ADE^+^ isolates tested for these strains, suggesting that the ADE^+^ phenotype is due predominantly or exclusively to the enhanced degradation observed for these chimeras (Figure 3B). Similar results were obtained for the Npl3 G-rich domain: insertion of four hydrophobic residues resulted in enhanced growth on SC-ade (Figure 4A, left) and increased the frequency of spontaneous prion formation (Figure 4A, right), whereas insertion of six hydrophobic residues led to a ~10,000–100,000-fold increase in growth with no reversion to the *ade*^−^ phenotype upon GuanHCl treatment. The WT G-rich LCDs in the context of the Sup35 PrD lie near the threshold for predicted prion activity, and insertion of hydrophobic residues increases their predicted prion propensity (Figure S4). These results indicate that: (1) both of the native G-rich LCDs are capable of supporting prion formation (albeit likely with involvement of the remainder of the Sup35 prion domain), (2) the frequency of prion formation is enhanced by insertion of residues predicted to enhance prion aggregation, and (3) prion formation occurs at a hydrophobicity threshold just below that which is robustly recognized by the proteostasis machinery.

Insertion of hydrophobic residues within the Q/N-rich LCDs from Rnq1 and Ure2 generally results in a progressive increase in the proportion of cells growing on SC-ade (Figure 4B, left), suggesting a progressive increase in the frequency of spontaneous prion formation. This effect is particularly striking for the Rnq1 Q/N-rich domain. Insertion of four hydrophobic residues resulted in a ~100-fold increase in growth on SC-ade, and insertion of six hydrophobic residues resulted in an additional ~1000-fold increase in growth. Contrary to the sharp transition in growth observed for the G-rich LCDs, all ADE^+^ isolates were curable with GuanHCl treatment (Figure 4B, right), indicating that prion formation rather than degradation was the primary cause of the ADE^+^ phenotype. The WT Ure2 Q/N-rich domain exhibited a higher frequency of spontaneous prion formation compared with the native Sup35 ND even before insertion of hydrophobic residues (Figure 4B; [31]). While the insertion of two hydrophobic residues in the Ure2 Q/N-rich LCD did not noticeably increase the frequency of spontaneous prion formation, insertion of four or six hydrophobic residues enhanced growth on SC-ade, indicating an increase in prion formation frequency. Finally, cells expressing the WT Q/N-rich domain from Ynl208W fused to Sup35 are phenotypically *ade*^−^, and insertion of up to four hydrophobic residues did not substantially enhance growth on SC-ade. However, insertion of six hydrophobic residues resulted in the appearance of a subset of ADE^+^ colonies that were cured by GuanHCl. Combined with the western blot data in Figure 3C, this suggests that the Ynl208W Q/N-rich domain similarly suppresses the degradation-promoting effects of hydrophobic residues and exhibits prion activity upon insertion of hydrophobic residues.

**Figure 5:**
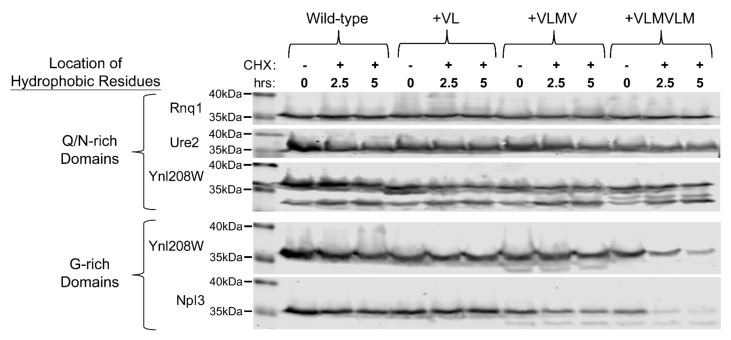
G-rich and Q/N-rich LCDs retain their respective susceptibility and resistance to hydrophobic degrons when fused to GFP. LCDs depicted in Figure 3 were N-terminally fused to GFP, and the stability (with respect to degradation) was monitored by western blot.

To further validate that the degradation susceptibility or resistance observed for the G-rich and Q/N-rich LCDs is an inherent feature of the domains (and not an artefact of the Sup35 fusion assay), we fused the LCDs to GFP and assayed the stability of the fusion proteins over time. All three Q/N-rich domains were stable over 5 h even with up to six hydrophobic residues inserted within the LCD (Figure 5), suggesting that these domains inherently tolerate the introduction of degrons without an increase in degradation rate. The Q/N-rich Ynl208W LCD showed two bands, suggesting that it may be subject to proteolytic cleavage, but neither of these two bands showed significant degradation, even with the insertion of up to six hydrophobic amino acids. In contrast, both of the G-rich LCDs exhibit an increase in degradation rate upon insertion of six hydrophobic residues (Figure 5), although the potency of the degrons in the Ynl208W G-rich domain appears to be lower in this context compared to in the PrLD-Sup35 fusions. These results suggest that susceptibility or resistance to hydrophobic degrons is an inherent property of G-rich and Q/N-rich domains, respectively.

### 2.3. Native Protein Context Influences Degradation Susceptibility of G-Rich and Q/N-Rich LCDs

We next asked whether the G-rich and Q/N-rich LCDs would be similarly susceptible or resistant to the degradation-promoting effects of hydrophobic insertions in their native protein contexts. Hydrophobic residues were inserted into each of the domains at sites identical to those indicated in Figure 3 in the context of their 2×HA-tagged parent proteins.

**Figure 6:**
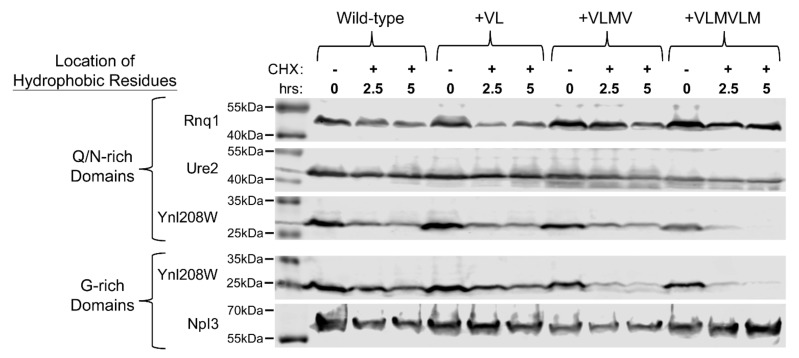
Sequence heuristics governing proteostatic regulation of G-rich and Q/N-rich LCDs exert mixed effects in the context of the full-length protein. The stability (with respect to degradation) of 2×HA-tagged, native G-rich and Q/N-rich proteins was monitored by western blot. Insertion of hydrophobic residues was performed in the same locations within the LCDs as those indicated in Figure 3.

As predicted, insertion of hydrophobic residues within the Q/N-rich LCDs of Rnq1 and Ure2 did not enhance degradation of the full-length proteins (Figure 6), suggesting that these Q/N-rich LCDs are capable of masking the degradation-promoting effects of hydrophobic residues in their native contexts.

Contrary to expectations, insertion of hydrophobic residues did not noticeably influence the degradation rate of Npl3. Npl3 is primarily involved in mRNA shuttling from the nucleus to the cytoplasm. Although Npl3 is exported into the cytoplasm as part of its normal function, it is predominantly observed in the nucleus [50]. To examine whether Npl3 localization to the nucleus suppresses its degradation when hydrophobic residues are inserted in the G-rich domain, the WT protein and Npl3 insertion mutants were tagged with a nuclear export signal (NES), which exerts a strong dominant effect over intrinsic nuclear localization sequences [51]. Additionally, Sky1 is a kinase that phosphorylates Npl3 and promotes its nuclear import: loss of Sky1 activity also enhances cytoplasmic localization of Npl3 [52]. Degradation of NES-Npl3 was assessed in both WT and sky1K187M (which catalytically inactivates Sky1 [53]) genetic backgrounds. In both genetic backgrounds, NES-Npl3 was also not degradation prone with up to six hydrophobic residues inserted (Figure S5), suggesting that degradation-promoting effects we typically observe for hydrophobic residues within G-rich LCDs are suppressed in the native Npl3 context.

Finally, Ynl208W allowed for direct comparison of the effects of hydrophobic insertion into Q/N-rich versus G-rich segments of the same protein. While WT Ynl208W is already somewhat degradation-prone over 5 h after addition of cycloheximide, insertion of four and six hydrophobic residues in the G-rich domain resulted in a significant increase in degradation rate (Figure 6 and Figure S6), which recapitulated the increase in degradation rate observed for the G-rich Ynl208W LCD fused to Sup35. Insertion of four and six hydrophobic residues into the Q/N-rich domain also significantly enhanced degradation: however, the degradation rate observed upon insertion of four hydrophobic residues within the Q/N-rich domain was significantly less than the degradation observed for the same insertion within the G-rich domain (*p* = 0.077 and 0.0026 at 2.5 h and 5 h post-CHX, respectively; Figure S6A). Thus, it appears that, although the Q/N-rich domain of Ynl208W is slightly more efficient than the G-rich domain at masking the degradation-promoting effects of hydrophobic amino acids in the context of full-length Ynl208W, this masking effect is more subtle than in the context of the fusion constructs. However, among the set of Q/N-rich LCDs tested, the Ynl208W Q/N-rich domain has the lowest Q/N content (see Q/N percentages in Figure 3C); it is possible that the threshold density of Q/N residues required to mask the degradation-promoting effects of hydrophobic degrons is context-dependent. Therefore, while the masking of degradation-promoting features appears to be a general property of Q/N-rich LCDs, the precise thresholds (both in terms of minimum hydrophobic content and minimum Q/N content) required for these effects may vary.

From these data, we conclude that the sensitivity/resistance of G-rich and Q/N-rich LCDs to the effects of hydrophobic degrons can (in some cases) be modulated by the native protein context, even though G-rich LCDs are unanimously sensitive to hydrophobic degrons and Q/N-rich LCDs are unanimously resistant to hydrophobic degrons when extricated from their native protein contexts. This is an important observation, as prediction methods based on model substrates are often extrapolated to native proteins without further validation. Therefore, additional experiments will be required to further elucidate the regulatory effects of neighboring protein regions in relevant proteins (see also Discussion).

### 2.4. Differential Sensitivity of Polar Low-Complexity Domains to Hydrophobic Degrons

To date, prior experiments have focused on the characterization of G-rich LCDs relative to mixed Q/N-rich LCDs. An interesting open question is whether Q-rich and N-rich LCDs will independently suppress hydrophobic degrons. Additionally, it is unclear whether other LCDs enriched in polar residues (namely S or T) will similarly suppress hydrophobic degrons or exhibit susceptibility more comparable to G-rich LCDs.

**Figure 7:**
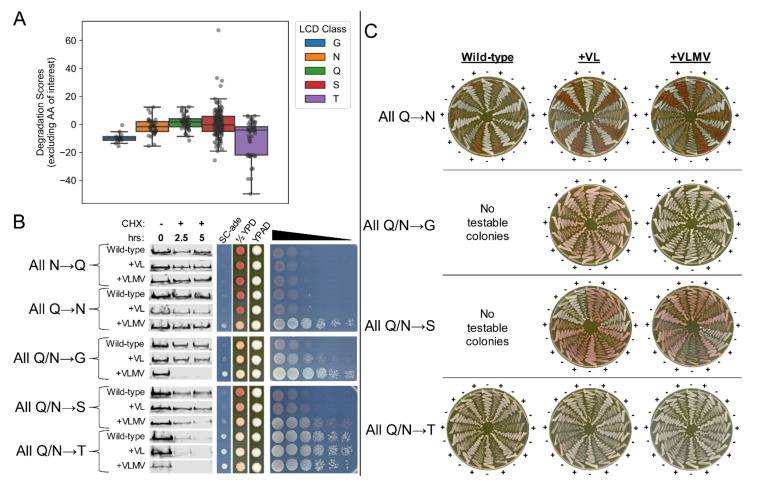
Compositional features of polar LCDs correspond to in vivo sensitivity to proteostatic degradation. (**A**) Degradation scores for G-rich and T-rich LCDs tend to be lower than degradation scores corresponding to Q-, N-, or S-rich LCDs. (**B**) Substitution of all Q residues in the native Sup35 ND for N or vice versa does not increase the sensitivity of the Sup35 ND to hydrophobic degrons (left) and generally results in an *ade*^−^ phenotype (right). Insertion of four hydrophobic residues results in the appearance of a prion-dependent *ADE*^+^ phenotype (see below). Substitution of all Q and N residues in the Sup35 ND for G, T, or S and subsequent insertion of hydrophobic residues accelerates degradation (left) and enhances the *ADE*^+^ phenotype (right). (**C**) *ADE*^+^ colonies corresponding to the Q→N substitution construct are curable by GuanHCl, indicative of a prion basis for the *ADE*^+^ phenotype. *ADE*^+^ colonies from the Q/N→G, Q/N→S, or Q/N→T are not curable by GuanHCl, consistent with enhanced degradation of these proteins. All N→Q substitution constructs yielded no viable colonies on SC-ade and were therefore omitted from panel C.

Figure 1 and Figure 2 collectively suggested that a selective pressure exerted on G-rich LCDs will tend to limit the number and clustering of hydrophobic residues, resulting in systematically lower degradation scores and higher protein half-lives. To explore whether this effect can be detected for any other polar LCD class independently (G, Q, N, S, or T), the yeast proteome was scanned separately for each class using LCD-Composer (window size = 40 aa, minimum composition threshold = 40%). Both Q-rich and N-rich LCDs exhibit higher degradation scores relative to G-rich LCDs (Figure 7A), suggesting that Q-rich and N-rich LCDs may independently suppress hydrophobic degrons. S-rich LCDs also exhibit high degradation scores, whereas a subset of T-rich LCDs exhibit extremely low degradation scores (Figure 7A), indicating that T-rich LCDs may also be susceptible to hydrophobic degrons.

To test these effects directly, all Q/N residues within the Sup35 ND were replaced with G, Q, N, S, or T, followed by insertion of zero, two, or four hydrophobic residues in each LCD. Substituting all Ns for Qs maintained the *ade*^−^ phenotype and resistance to degradation (Figure 7B and Figure S7), with no apparent increase in prion formation even with insertion of up to four hydrophobic residues (as evidenced by a complete lack of growth on SC-ade). Substituting all Qs for Ns typically resulted in an *ade*^−^ phenotype and degradation resistance (Figure 7B). However, rare ADE^+^ colonies for the WT (i.e., no hydrophobic residues inserted) and the +VL insertion in the N-rich ND were curable (Figure 7C), indicating that Q→N substitutions enhance prion propensity, consistent with previous observations [54]. Additionally, the +VLMV insertion resulted in a robust and consistently curable ADE^+^ phenotype (Figure 7C) with no apparent increase in degradation rate (Figure 7B).

In contrast, the G-rich ND (all Q/Ns substituted for G) resulted in a weak ADE^+^ phenotype that was progressively enhanced upon insertion of two and four hydrophobic residues (Figure 7B), and ADE^+^ colonies were not cured by GuanHCl (Figure 7C). While insertion of two hydrophobic residues in the G-rich ND did not noticeably influence its degradation rate, insertion of four hydrophobic residues resulted in rapid degradation (Figure 7B), consistent with our working model and the robust ADE^+^ phenotype. The S-rich ND exhibited an *ade*^−^ phenotype in the absence of insertions. Insertion of two or four hydrophobic residues resulted in a weak ADE^+^ phenotype that is slow-growing, incurable, and unusually pink on SC-ade media (Figure 7B,C). Furthermore, the S-rich ND was only mildly susceptible to degradation upon insertion of four hydrophobic residues (Figure 7B), consistent with partial degron suppression activity. By comparison, the T-rich ND was remarkably susceptible to degradation: it was partially degraded even in the absence of hydrophobic insertions, this effect was enhanced upon insertion of hydrophobic residues, and all three strains exhibited a robust ADE^+^ phenotype that was not cured by GuanHCl (Figure 7B,C). It should be noted that lower molecular weight products are specifically produced for the N-rich ND with two hydrophobic residues inserted and the T-rich ND with four hydrophobic residues inserted, although these do not appear to influence the degradation rates of the full-length proteins (Figure S7).

In combination with Figure 7A, this suggests that T-rich LCDs (but not S-rich LCDs) may also be subject to selective pressures that limit their degradation propensity in native proteins.

## 3. Discussion

Sufficient proteostasis is a fundamental requirement of life. However, maintaining proteostasis is no small feat: a remarkable diversity of proteins with unique sequences, states, and functions are required for even the simplest forms of life. Consequently, cells possess hundreds or thousands of proteins that work in a coordinated fashion to maintain cellular proteostasis. The prevention of aberrant protein aggregation is perhaps one of the most important yet formidable challenges assigned to the proteostasis machinery, requiring an intricate balance between sensitive detection of aggregation-prone features and adequate permissiveness to tolerate the diversity of protein sequences and folds required by the cell. Many proteostasis factors specifically recognize aggregation-prone features and attempt to re-fold or degrade the protein. While a high aggregation propensity is likely a prerequisite for protein aggregation in vivo, it may not be entirely sufficient given the perpetual activity of working proteostasis systems.

Our model G-rich and Q/N-rich LCDs [31] facilitated the initial discovery and characterization of these regulatory principles. Here, we examined whether these heuristics generalize to other native G-rich and Q/N-rich LCDs. Insertion of hydrophobic residues into two native yeast G-rich LCDs increased their degradation rate, as observed previously for the G-rich hnRNP PrLDs. Likewise, insertion of hydrophobic residues into two native Q/N-rich LCDs from other known yeast prion proteins had no detectable effect on degradation rate, and instead led to an increase in the frequency of spontaneous prion aggregation, consistent with analogous experiments in the Q/N-rich Sup35 PrD. Furthermore, insertion of hydrophobic residues into the Q/N-rich domain of Ynl208W also had no effect on its degradation rate but promoted prion aggregation at sufficiently high hydrophobic content when substituted in place of the Sup35 ND. Importantly, prion aggregation of Ynl208W protein has never been observed for the WT protein, suggesting that insertion of hydrophobic residues exerts consistent effects even in a Q/N-rich domain with no known prion activity.

When combined with our previous work [31], we have tested a variety of native LCDs, heterologous LCDs, and artificial/synthetic LCDs. In all cases, G-rich LCDs exhibit sensitivity to hydrophobic degrons, whereas Q/N-rich LCDs exhibit resistance to hydrophobic degrons when removed from their native protein context (i.e., either fused to Sup35 or GFP). However, an important conclusion from these experimental results is that, while the sequence features governing proteostatic regulation of G-rich and Q/N-rich LCDs have thus far generalized perfectly to native yeast LCD fragments in isolation, they did not perfectly predict the behavior of analogous mutations in the context of their native proteins. This is particularly important since, analogous to our Sup35 reporter assay, isolated protein fragments are commonly used to initially define regulatory principles, which are then subsequently applied to full-length proteins in their native contexts. Indeed, our data are consistent with numerous previous studies indicating that relationships between protein sequence and degradation rates are rarely absolute on a proteome-wide scale, and degradation rate for a given protein likely results from the culmination and integration of multiple regulatory signals [55,56,57,58,59,60]. Therefore, other factors such as protein localization, protein-protein interaction, or neighboring protein sequences could influence the proteostatic regulation of proteins containing G-rich and Q/N-rich LCDs. Subcellular localization of proteins may be particularly important, as subcellular compartments often exhibit unique, compartment-specific proteostatic regulation [2] and different rates of degradation for the same protein when targeted to different subcellular compartments [61]. This may be especially relevant for T-rich LCDs: the T-rich variant of Sup35 (a cytoplasmic protein) was highly degradation-prone even in the absence of hydrophobic insertions (Figure 7), yet proteins with T-rich LCDs are almost entirely excluded from the cytoplasm (Figure S8 and Table S4) and are strongly associated with the cell periphery [14,32]. Therefore, we speculate that the proteostasis machinery may not only select against specific features within LCDs but also might select against entire classes of LCDs. However, future studies are required to extensively validate this hypothesis.

To date, our studies have focused on uncovering the features of polar LCDs that lead to their susceptibility to, or suppression of, hydrophobic degrons. However, we have not yet explored possible mechanisms responsible for these behaviors, and we can only speculate on how or why (evolutionarily) these classes of LCDs diverge with respect to proteostatic regulation. In general, proteostasis factors recognize protein features that, if left unchecked, tend to have deleterious effects in the cell (e.g., improper folding or unfolding of essential proteins, damage to existing proteins, and aggregation). It is possible that degradation-sensitive classes of LCDs are at a greater risk of becoming toxic unless they are cleared or chaperoned efficiently by the proteostasis machinery. Presumably, the differential sensitivity is likely a function of specific binding sites among the proteostasis factors that initially recognize the LCDs, the structural behavior of the LCDs, or some combination of the two. Highly polar segments tend to favor intrinsic disorder. However, the conformational ensembles populated by intrinsically disordered domains can differ substantially depending on sequence and composition [62]. In particular, computational and experimental results suggest that G-rich and Q/N-rich LCDs may form highly dynamic collapsed ensembles that remain disordered [62], preferring self-solvation over solvation with water. If this activity generalizes to our LCDs, hydrophobic degrons would be required to partition between these “solvents”. In such a scenario, hydrophobic degrons may partition to the interior of collapsed Q/N-rich LCDs (effectively “hiding” them from surrounding molecules) but inadequately partition to the interior of collapsed G-rich LCDs, either due to exclusion from the interior of the collapsed polypeptide or incomplete “hiding” of the hydrophobic surface area by the relative paucity of large sidechains. Alternatively, it is possible that Q/N-rich segments sample structured conformations for longer periods of time or favor more collapsed (rather than extended) disordered conformations relative to G-rich LCDs, modulating the exposure time of hydrophobic degrons. However, we again emphasize that these models are highly speculative and require future experimentation to test.

One of the fundamental challenges faced by the proteostasis machinery is the balance between sensitivity and specificity. Proteostasis factors must be able to accurately detect abnormal or misfolded proteins without interfering with the production of healthy proteins. We have previously observed that insertion of as few as two hydrophobic residues within G-rich PrLDs is sufficient to accelerate degradation of the protein [31]. While the threshold for the number of hydrophobic residues required to enhance degradation varies among G-rich PrLDs, prion aggregation is observed for insertions just below this threshold for both G-rich PrLDs examined here. Therefore, the trade-off between proteostatic sensitivity and specificity may result in a proteostasis sensitivity gap (as previously proposed [36]), allowing some proteins to aggregate via a combination of sufficient aggregation propensity and low detectability by proteostasis factors. It is worth pointing out that evasion of the proteostasis machinery need not be absolute in order for some proteins to ultimately form persistent aggregates in vivo: the propagation of yeast prion aggregates actually depends on the disaggregase Hsp104 (and associated co-chaperones; [63]). While Hsp104 successfully eliminates certain structural prion polymorphs (e.g., [64,65]), others are incompletely processed by Hsp104, resulting in prion aggregate fragmentation (and, thus, propagation) rather than aggregate elimination. Additionally, the yeast prion, [*PSI*^+^], is cured upon overexpression of Hsp104, suggesting that an increase in Hsp104 activity can be sufficient for complete dissolution (rather than simply fragmentation) of prion aggregates [66]. From this perspective, while the Hsp104 chaperone system appears to play a positive role in prion propagation, this may also be viewed simply as inefficient recognition or inadequate disaggregase activity by Hsp104, resulting in a competitive advantage by certain protein aggregates that allows them to arise, persist, and propagate.

Finally, the constant surveillance of proteostasis factors effectively determines the survival time for each type of protein, constituting a form of natural selection at the molecular level. Therefore, protein sequence landscapes observed in unmodified organisms have necessarily survived this form of molecular selection. Our experimental evidence indicating that the features controlling the fates of different PrLDs extend broadly to G-rich and Q/N-rich LCDs, coupled with bioinformatic sequence analysis of complete proteomes, suggest that the allowable sequence space for G-rich and Q/N-rich LCDs is influenced by the constraints imposed upon them by the proteostasis machinery. Native G-rich LCDs from yeast and human proteins tend to have fewer potential degrons compared to native Q/N-rich LCDs. The avoidance of degrons within G-rich LCDs corresponds to longer half-lives for G-rich proteins in both yeast and humans, suggesting that this constitutes a molecular strategy for adapting to proteostatic constraints and achieving sufficient stability to perform necessary cellular functions. Finally, the compositional features of polar LCDs generally correlate with experimentally determined protein stability, indicating that similar selective pressures may be exerted on LCDs, with varying degrees of susceptibility between classes. However, we also demonstrate that LCDs in their native protein contexts do not always exhibit the effects predicted by their behavior in isolation, suggesting that neighboring protein features can provide an additional opportunity for proteins to modulate the inherent susceptibility/resistance of LCDs to degradation.

In short, these results provide key insight into the proteostatic fates of G-rich and Q/N-rich LCDs on a broad scale, and suggest that established proteostasis systems impose constraints on the allowable sequence space that can be explored by LCDs in multiple eukaryotic organisms.

## 4. Materials and Methods

### 4.1. Yeast Strains, Media, and Growth Conditions

Yeast growth conditions were as previously described [31,67]. Briefly, standard media components and concentrations were used, with two exceptions: YPD contained half of the standard amount of yeast extract (0.5% final concentration), and YPAD contained the standard amount of yeast (1%) extract supplemented with 0.02% adenine hemisulfate [yeast extract (product #Y20020-500.0) and peptone (product #P20250-1000.0)—Research Products International, Mount Prospect, IL, USA; Agar (product #214010)—BD Biosciences, Franklin Lakes, NJ, USA; adenine hemisulfate (product #A9126-25G)—Sigma-Aldrich, St. Louis, MO, USA]. Yeast strains used in this study were YER826/pER589 (*α kar1-1 SUQ5 ade2-1 his3 leu2 trp1 ura3 sup35::KanMx*) and YER279 (*α kar1 SUQ5 ade2-1 his3 leu2 trp1 ura3*). pER589, which expresses a truncated version of Sup35 to maintain viability, was replaced via plasmid shuffling with each of the LCD-Sup35 fusions to assess Sup35 activity using standard red/white phenotypic assays in an *ade2-1* genetic background. Mutagenesis of the hnRNP PrLDs, phenotypic screening, and determination of degradation-propensity scores for each amino acid was performed as previously described [31]. When necessary, SC-ade phenotype plates (Figure 4 and Figure 7B) were grown for an additional day to obtain colonies of sufficient size to test for curability.

### 4.2. Selection and Mutation of Native Yeast G-Rich and Q/N-Rich LCDs

We identified LCDs from native yeast proteins that were at least 50 aa in length and with G or Q/N compositions resembling those within the hnRNP PrLDs (~35% G) and Sup35ND (~50% Q/N), respectively, using the LCD-Composer algorithm [14]. LCDs approximately 50 aa in length were then manually selected for the Sup35 fusion and GFP fusion assays. In addition to LCDs identified by these criteria, we noticed a moderately Q/N-rich domain within one of the candidate G-rich proteins, Ynl208W. Although the Q/N-content for this domain was slightly lower than the Q/N content of the Sup35 ND (and the other Q/N-rich LCDs tested), we included it in the dataset in order to directly compare the contextual effects of a G-rich domain and a Q/N-rich domain from the same protein.

For targeted insertion of hydrophobic residues within native G-rich and Q/N-rich LCDs, insertions were incorporated in the center of each LCD using mutagenic oligonucleotides during PCR amplification for the LCD-Sup35 fusions and in the context of their parent proteins. To assay protein degradation for the native G-rich and Q/N-rich proteins, a 2×HA tag was added to the terminus opposite the LCD with one exception: for consistency, Ynl208W was tagged at the N-terminus for hydrophobic insertions within both the N-terminal Q/N-rich domain and the C-terminal G-rich domain [a C-terminal 2×HA tag on Ynl208W induced aberrant proteolytic cleavage of the protein in the absence of hydrophobic insertions (data not shown)].

### 4.3. Degradation Assays

Protein degradation was assessed as previously described [31], with minor differences. Briefly, strains were grown in liquid YPAD overnight at 30 °C with shaking, then diluted to a starting OD_595_ of 0.75 and grown for 1hr prior to adding CHX (50 µg/mL final concentration; VWR International, LLC., Radnor, PA, USA, product #97064-724). After the treatment period, optical density was used to approximate cell concentrations and normalize the total amount of cells harvested within each strain to the least-dense culture. Cells were harvested and lysed as described in [8,31]. 17 µL of lysate were loaded onto a polyacrylamide gel, transferred to a PVDF membrane, and blotted with the appropriate primary antibody. LCD-Sup35 fusions were probed with an anti-Sup35C monoclonal antibody (E4 [68], a gift from Susan Liebman), LCD-GFP fusions were probed with an anti-GFP monoclonal antibody (Santa Cruz Biotechnology, Inc., Dallax, TX, USA, product #sc-9996), and native G-rich and Q/N-rich proteins possessing a 2×HA tag were probed with an anti-HA monoclonal antibody (BioLegend, San Diego, CA, USA, product #901502). For all blots, a fluorophore-conjugated, polyclonal goat anti-mouse IgG secondary antibody was used (LI-COR, Lincoln, NE, USA, product #926-32210).

### 4.4. Statistics, Bioinformatics, and Data Sources

Yeast protein reference sequences were derived from the translated ORF proteome (from the Saccharomyces Genome Database website, last modified 13 January 2015). Human protein reference sequences were downloaded from the UniProt website (organismID: UP000005640_9606) on 22 February 2020.

To identify protein regions resembling those of the hnRNP PrLDs (≥35% G composition) and Sup35ND (≥50% Q/N composition), the yeast proteome was scanned using LCD-Composer [14] with both a short (20 aa) window and long (40 aa) window size. Whole-LCD degradation scores were defined as the sum of the degradation propensities for all amino acids, excluding the LCD-defining amino acids (G for G-rich LCDs, and Q and N for Q/N-rich LCDs). For clustering analyses, each G-rich or Q/N-rich LCD was subsequently scanned using a 8 aa or 4 aa scanning window, corresponding to the full length or ½ the length, respectively, of the mutagenized region in our genetic screen. The maximum sum total score for each LCD (again excluding the LCD-defining amino acid) was calculated. To compare the maximum cluster scores to naïve expectation, the sequence of each LCD was independently scrambled 10k times. The maximum cluster score for each scrambled variant was calculated as described above, then averaged across all 10k scrambling iterations to yield a paired, composition-matched score for each LCD. To facilitate direct comparison of polar LCDs generally, the yeast proteome was re-scanned using LCD-Composer with a 40% composition threshold and 40 aa window size for G, Q, N, S, or T separately. Additionally, a strict 40 aa minimum LCD length requirement was imposed after LCD identification to more closely reflect the original hnRNP PrLDs and Sup35 ND.

To generate composition plots (appearing Figure 3A), we developed a customizable Python script (CompositionPlotter) to visualize the local amino acid composition of an amino acid or group of amino acids for individual proteins. Selected proteins were scanned using a 40 aa window size for G and/or combined Q/N content. CompositionPlotter is available at (https://github.com/RossLabCSU/IJMS_2021, accessed on 19 April 2021).

Prion propensity predictions for the G-rich and Q/N-rich LCDs fused to Sup35 were performed using the downloadable modified prion aggregation prediction algorithm (mPAPA) Python script (https://github.com/RossLabCSU/mPAPA, accessed on 26 April 2021; [21,24]). By default, mPAPA uses FoldIndex to identify intrinsically disordered regions and only evaluates disordered domains for prion propensity. All of our model LCDs are initially classified as disordered. In some cases, insertion of hydrophobic residues into our model LCDs resulted in re-classification from disordered to ordered: therefore, for consistency across mutants, LCD sequences were evaluated without a requirement that the domain is scored as disordered. For each fusion protein, the full LCD-Sup35 fusion sequence was used in the prediction. For simplicity of interpretation, mPAPA scores corresponding only to the LCD region are indicated in Figure S4.

Protein half-lives for yeast and human proteins were derived from [37] and [38], respectively. Ambiguous yeast protein half-life annotations were discarded as detailed in [32]. For human proteins, all isoforms contained in the half-life dataset and the reference human proteome were retained in the analysis.

Degradation scores for the amino acid clustering analyses (Figure 2C,D) were approximately normal and statistically compared using a paired, two-tailed Student’s *t*-test. However, values for protein half-lives and for predicted degradation scores within native G-rich and Q/N-rich LCDs were often non-normally distributed. Therefore, for consistency and to facilitate direct comparisons, all other statistical comparisons employed the non-parametric two-tailed Mann–Whitney *U* test. Statistics and plotting were performed using the SciPy and Matplotlib/Seaborn packages, respectively, implemented in custom Python scripts.

## 5. Conclusions

In vivo, protein aggregation occurs in the context of extensive proteostasis machinery. In this study, we further defined features of polar LCDs that lead to sensitivity or resistance to proteasome-mediated degradation and differential propensity for prion aggregation. Specifically, G-rich, T-rich, and S-rich LCDs are susceptible to hydrophobic degrons to varying degrees, whereas Q-rich and N-rich LCDs display resistance to hydrophobic degrons and instead often form prion aggregates. However, these regulatory principles can, in some cases, be modulated by other regions in their native proteins. Finally, post hoc bioinformatic analyses suggest that the proteostasis machinery may impose a selective constraint on allowable sequence space for certain types of LCDs.

## Data Availability

The data presented in this study are available in this article and associated Supplementary Material. All code required to reproduce bioinformatic analyses is available at https://github.com/RossLabCSU/IJMS_2021, accessed on 19 April 2021.

## Supplementary Materials

The following are available online at https://www.mdpi.com/article/10.3390/ijms22168944/s1, Figure S1: Degradation scores for the canonical amino acids are highly correlated between the hnRNPA1 and hnRNPA2 libraries; Figure S2: Smaller clusters (4 aa window) do not dramatically affect trends in degron scores within G-rich and Q/N-rich LCDs; Figure S3: Western blots of LCD-Sup35 fusions with molecular weight markers; Figure S4: Predicted prion propensity of native G-rich and Q/N-rich LCDs with insertion of hydrophobic residues, in the context of the Sup35 fusion; Figure S5: Redistributing full-length Npl3 to the cytoplasm does not alter its sensitivity to degradation upon hydrophobic insertion; Figure S6: Quantification of full-length Ynl208W upon insertion of hydrophobic residues in the G-rich or Q/N-rich LCD; Figure S7: Western blots of polar Sup35 LCD substitution variants with molecular weight markers; Figure S8: Subcellular localization frequencies for proteins with polar LCDs; Table S1: Protein half-lives for yeast and human proteins containing G-rich or Q/N-rich LCDs; Table S2: G-rich and Q/N-rich LCD sequences in yeast and humans; Table S3: Degradation scores for the canonical amino acids from the expanded hnRNPA1 and hnRNPA2 libraries; Table S4: Subcellular localization frequencies for proteins containing polar LCDs.
