# Supplementary material for: Generalizable Compositional Features Influencing the Proteostatic Fates of Polar Low-Complexity Domains
Source: Int J Mol Sci. 2021 Aug 19;22(16):8944. doi: 10.3390/ijms22168944 (PMC8396281; doi:10.3390/ijms22168944)
Supplement: Supplementary file 1 [file ijms-22-08944-s001.zip › Supplementary Figures and Supplementary Table Legends_Revision.pdf]

Supplementary Figures

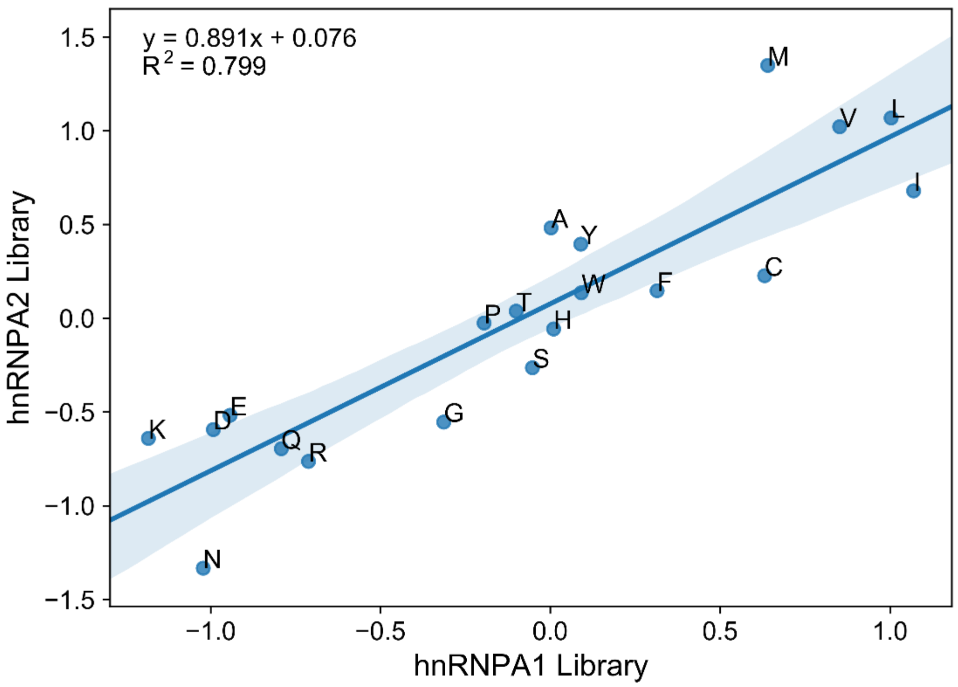

Fig S1. Degradation scores for the canonical amino acids are highly correlated between the hnRNPA1 and hnRNPA2 libraries. Ordinary least squares regression was performed for the degradation scores (Table S3) derived from the hnRNPA1 and hnRNPA2 libraries.

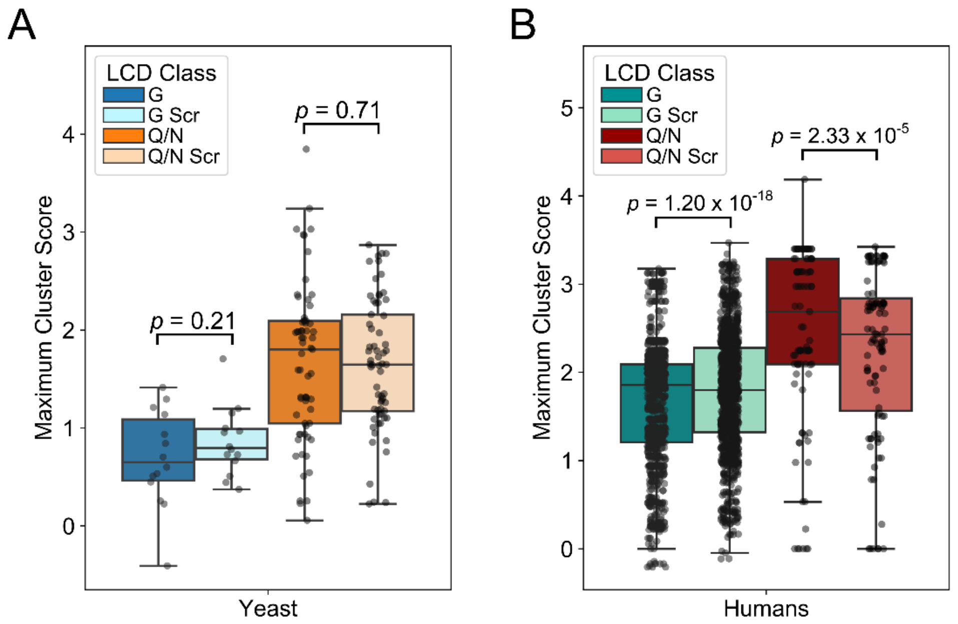

Fig S2. Smaller clusters (4aa window) do not dramatically affect trends in degron scores within G-rich and Q/N-rich LCDs. Clustering analyses were performed as indicated for Fig 2C,D, except that a 4aa sliding window was used. (A) Scrambled yeast G-rich LCDs tend to have slightly higher (though not statistically significant) maximum cluster scores than their native counterparts (two-sided, paired *t*-test; see Materials and Methods). (B) Scrambled human G-rich LCDs have similar or slightly higher maximum cluster scores than native G-rich LCDs, whereas scrambled Q/N-rich LCDs have significantly lower maximum cluster scores relative to their native counterparts.

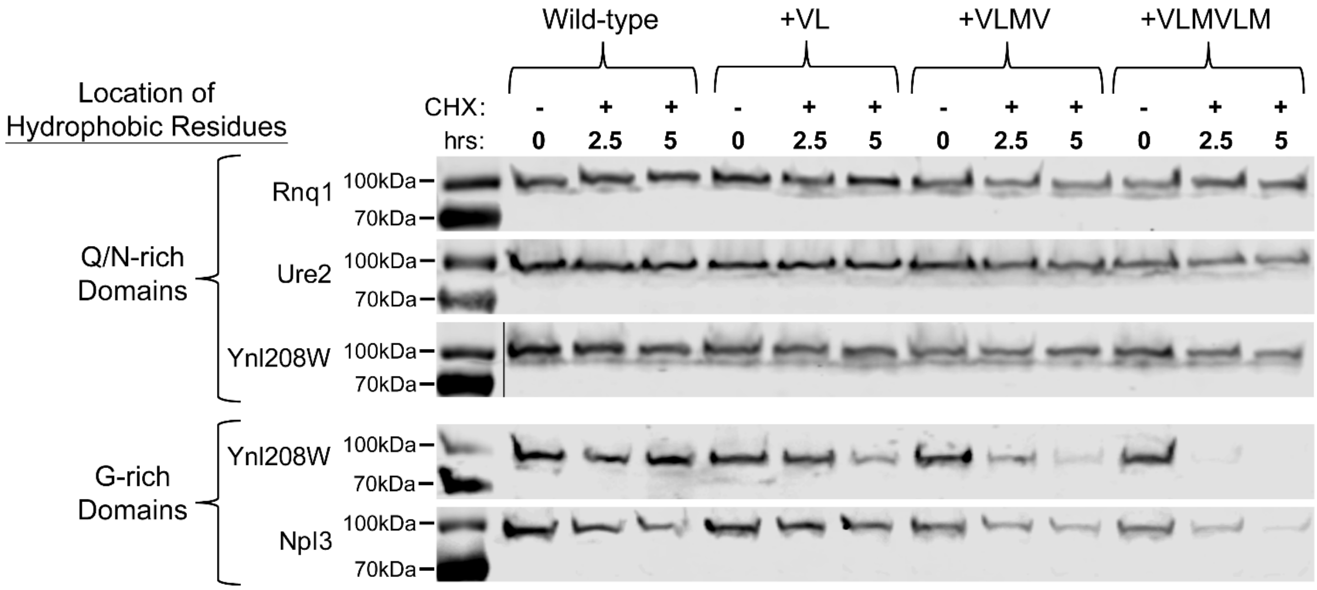

Fig S3. Western blots of LCD-Sup35 fusions with molecular weight markers. Blots are identical to those in Fig 3, but with molecular weight markers shown.

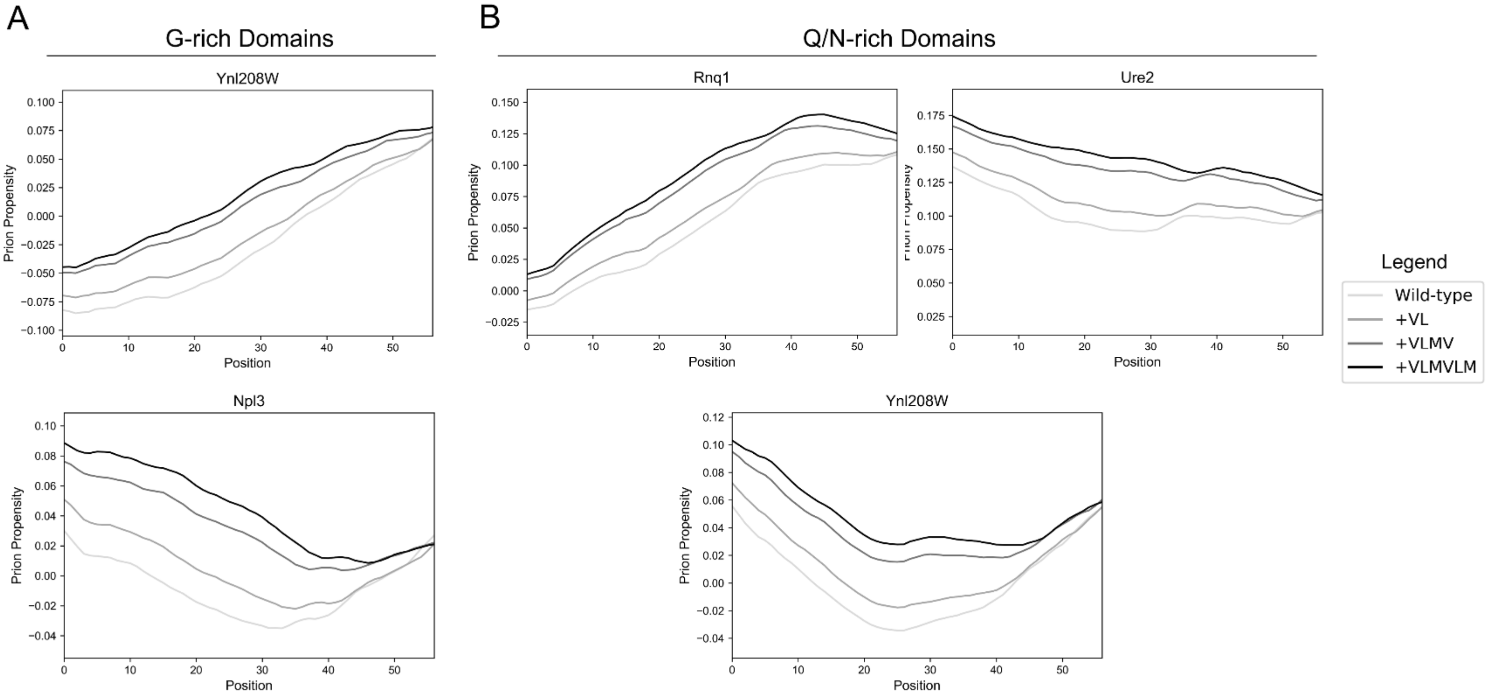

Fig S4. Predicted prion propensity of native G-rich and Q/N-rich LCDs with insertion of hydrophobic residues, in the context of the Sup35 fusion. Sequences of the native G-rich and Q/N-rich LCDs substituted in place of the Sup35 nucleation domain, which correspond to those tested in Figs 3-4, were analyzed using the modified prion aggregation prediction algorithm (mPAPA) [1,2]. Only prion propensities corresponding to the G-rich or Q/N-rich regions of interest are depicted. (A) Insertion of two hydrophobic residues in the Ynl208W G-rich domain leads to a mild increase in predicted prion propensity (consistent with rare prion formation observed for this mutant; Fig 4), while insertion of four or six hydrophobic residues leads to a sharper increase in predicted prion propensity, which appears to be efficiently detected by the proteostasis machinery (Fig 3). Likewise, insertion of six hydrophobic residues in the G-rich domain of Npl3 leads to a large increase in predicted prion propensity but also to efficient detection by the proteostasis machinery (Fig 3), whereas insertion of four hydrophobic residues increases predicted prion propensity but lies below the threshold of detection by the proteostasis machinery (Figs 3,4). (B) Insertion of hydrophobic residues consistently leads to an increase in predicted prion propensity and high absolute prion propensity scores for Rnq1 and Ure2, whereas the absolute prion propensity scores for the majority of the Ynl208W Q/N-rich domain remain relatively low.

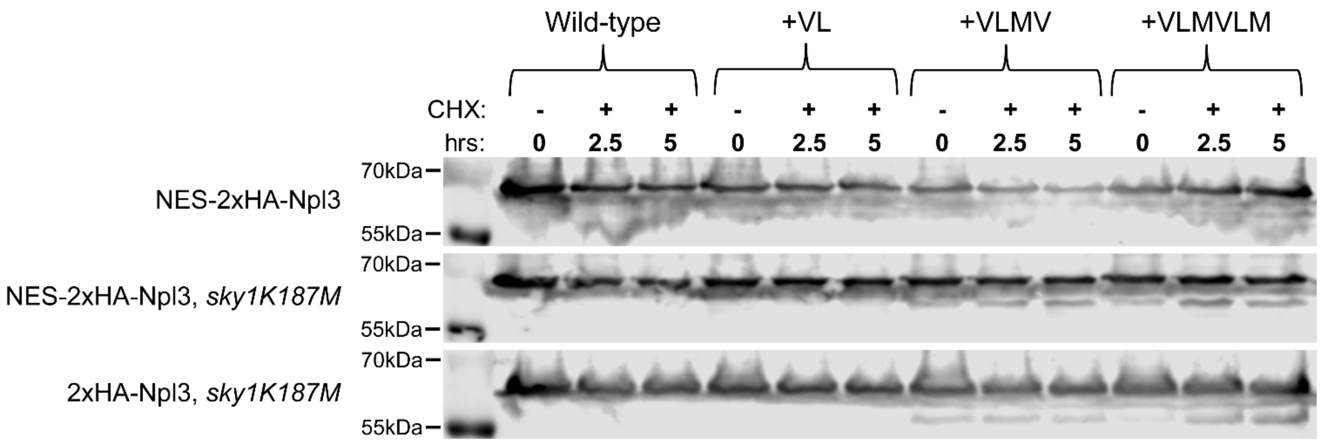

Fig S5. Redistributing full-length Npl3 to the cytoplasm does not alter its sensitivity to degradation upon hydrophobic insertion. Progressive insertion of hydrophobic residues into the G-rich domain of Npl3 tagged with a nuclear export signal (NES; *top*), in a *sky1K187M* genetic background (*bottom*), or with an NES in a *sky1K187M* background (*middle*) does not increase its rate of degradation. However, a slight amount of low molecular weight products are observed for the +4 and +6 insertions (particularly in the *sky1K187M* background), potentially indicating an increase in site-specific proteolytic cleavage dependent upon the degree of hydrophobicity.

A

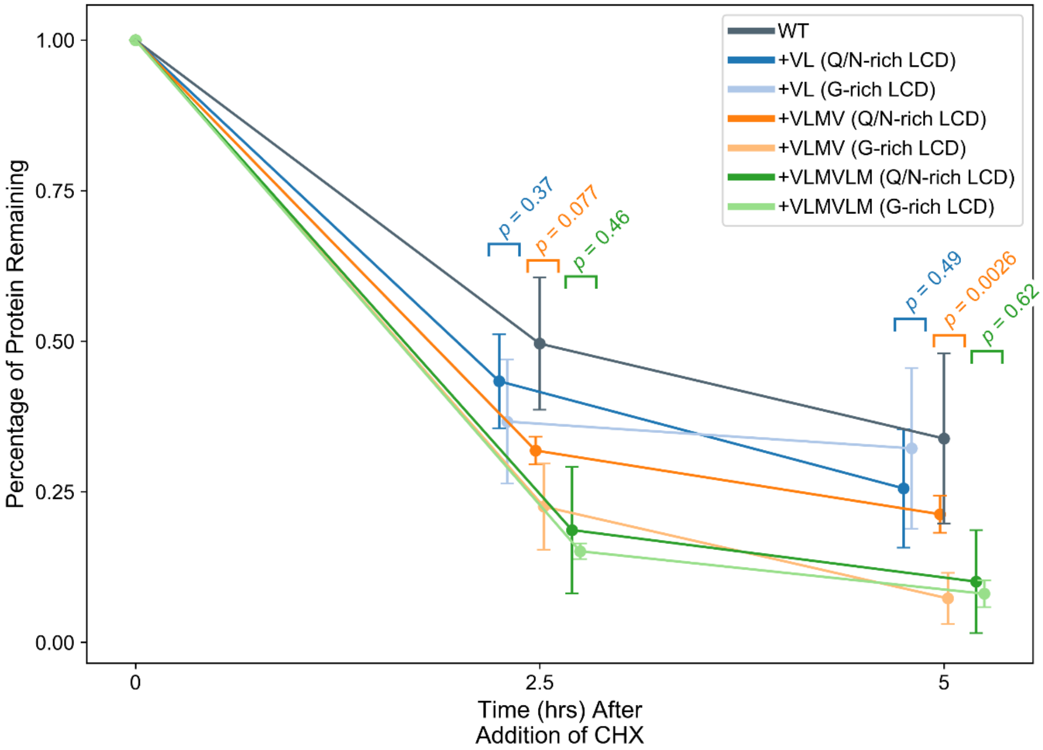

B

Statistical Comparisons Relative to WT

Location of Hydrophobic Residues	Hydrophobic Insertion	2.5hr Timepoint	5hr Timepoint
Q/N-rich LCD	+VL	p = 0.40	p = 0.38
	+VLMV	p = 0.025	p = 0.17
	+VLMVLM	$p = 2.3 \times 10^{-3}$	p = 0.025
G-rich LCD	+VL	p = 0.058	p = 0.84
	+VLMV	$p = 5.0 \times 10^{-4}$	$p = 2.0 \times 10^{-3}$
	+VLMVLM	$p = 2.7 \times 10^{-5}$	$p = 2.1 \times 10^{-3}$

Fig S6. Quantification of full-length Ynl208W upon insertion of hydrophobic residues in the G-rich or Q/N-rich LCD. (A) At least 3 experimental replicates were performed for the Ynl208W full-length protein with insertion of hydrophobic residues in either the moderately Q/N-rich LCD or the G-rich LCD, as described in Fig 6. The percentage of protein remaining for each Ynl208W variant was calculated as the normalized fluorescence relative to the same strain in the absence of CHX (0hrs time point). Scatter points represent the average normalized intensity (± s.d.) across experimental replicates. Scatter points are slightly shifted on the x-axis at the 2.5hrs and 5hrs time points for visual clarity only. Paired colors depict each hydrophobic insertion (e.g. +VL) within the Q/N-rich LCD (dark color) and G-rich LCD (corresponding light color). For each pair, normalized intensity values were statistically compared using a two-sided Student's *t*-test at each timepoint, with color-coded *p*-values indicated above each pair. (B) For each hydrophobic insertion, normalized intensity values were compared to those of the wild-type (WT) protein at the corresponding timepoint using a two-sided Student's *t*-test.

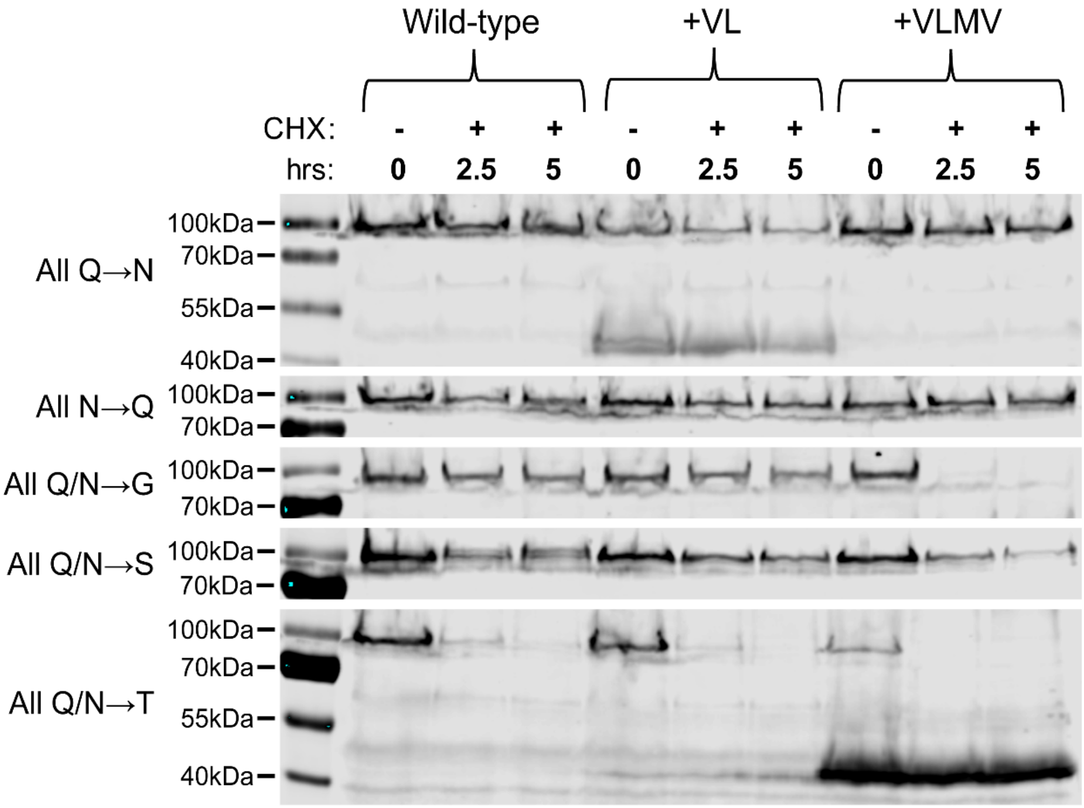

Fig S7. Western blots of polar Sup35 LCD substitution variants with molecular weight markers. Blots are identical to those in Fig 7, but with molecular weight markers shown. Additionally, lower molecular weight products are apparent for the Q→N +VL construct and the Q/N→T +VLMV construct, which presumably represent proteolytic cleavage products specifically generated for these sequences but do not appear to affect degradation rates of the full-length proteins.

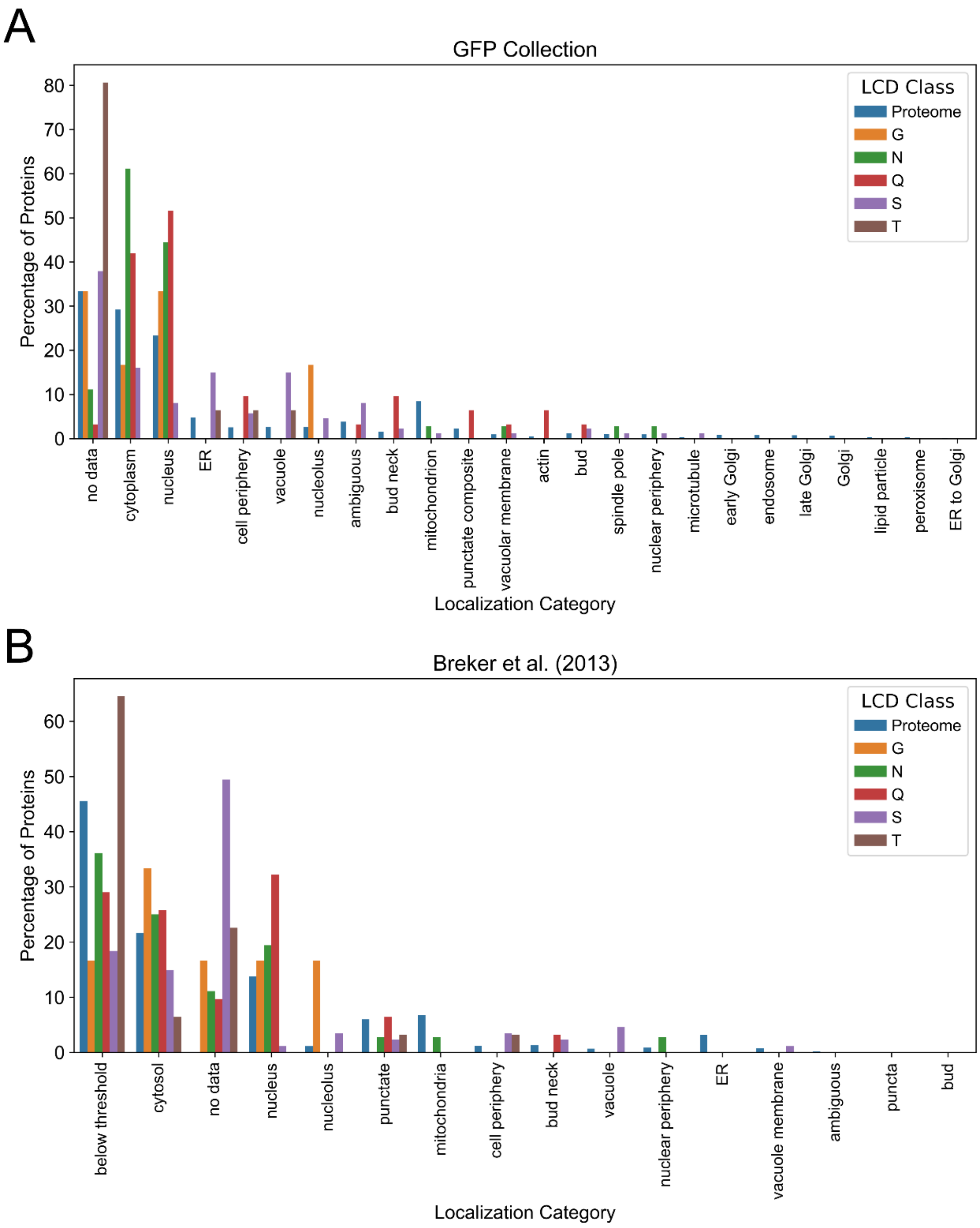

Fig S8. Subcellular localization frequencies for proteins with polar LCDs. For each LCD class, all proteins containing an LCD at least 50aa long were cross-referenced against two subcellular localization databases: (A) the classic yeast GFP collection [3], and (B) the dataset by Breker *et al.* [4]. The "Proteome" class consists of all yeast proteins and was included for comparison with yeast LCD-containing proteins. The percentage of proteins annotated with each localization category was calculated independently for each localization category: consequently, since some proteins are associated with multiple subcellular compartments, the sum of percentages across localization categories for each LCD class (or "Proteome") may exceed 100.

Supplementary Table Legends

Table S1. Protein half-lives for yeast and human proteins containing G-rich or Q/N-rich LCDs. LCDs were identified using LCD-Composer with minimum composition thresholds of 35% and 50% for G-rich or Q/N-rich LCDs, respectively. Subsequent length filters were applied to attain proteins with short (≥ 20aa) or long (≥ 40aa) LCDs. Protein half-lives were derived from [5] and [6] for yeast and human proteins, respectively.

Table S2. G-rich and Q/N-rich LCD sequences in yeast and humans. Complete list of LCD sequences and corresponding amino acid compositions for long LCDs (≥ 40aa) identified by LCD-Composer (see Table S1 and Materials and Methods).

Table S3. Degradation scores for the canonical amino acids from the expanded hnRNPA1 and hnRNPA2 libraries. Experimental derivation and calculation of degradation scores was performed as detailed in [7]. To generate a single set of degradation propensity scores, the amino acid frequencies from the hnRNPA1 and hnRNPA2 libraries were first summed before calculating the corresponding odds ratios. Degradation scores were defined as the natural logarithm of the odds ratio for each amino acid using the combined frequencies from the hnRNPA1 and hnRNPA2 libraries.

Table S4. Subcellular localization frequencies for proteins containing polar LCDs. Subcellular localization percentages were calculated as described for Fig S7.

References

1. Toombs, J.A.; McCarty, B.R.; Ross, E.D. Compositional determinants of prion formation in yeast. *Mol. Cell. Biol.* **2010**, *30*, 319–332.
2. Cascarina, S.M.; Ross, E.D. Natural and Pathogenic Protein Sequence Variation Affecting Prion-Like Domains Within and Across Human Proteomes. *BMC Genomics* **2020**, *21*, doi:10.1186/s12864-019-6425-3.
3. WK, H.; JV, F.; LC, G.; AS, C.; RW, H.; JS, W.; EK, O. Global analysis of protein localization in budding yeast. *Nature* **2003**, *425*, 686–691, doi:10.1038/NATURE02026.
4. Breker, M.; Gymrek, M.; Schuldiner, M. A novel single-cell screening platform reveals proteome plasticity during yeast stress responses. *J. Cell Biol.* **2013**, *200*, 839–850, doi:10.1083/jcb.201301120.
5. Christiano, R.; Nagaraj, N.; Fröhlich, F.; Walther, T.C. Global Proteome Turnover Analyses of the Yeasts S.cerevisiae and S.pombe. *Cell Rep.* **2014**, *9*, 1959–1966, doi:10.1016/j.celrep.2014.10.065.
6. Cambridge, S.B.; Gnad, F.; Nguyen, C.; Bermejo, J.L.; Krüger, M.; Mann, M. Systems-wide proteomic analysis in mammalian cells reveals conserved, functional protein turnover. *J. Proteome Res.* **2011**, doi:10.1021/pr101183k.
7. Cascarina, S.M.; Paul, K.R.; Machihara, S.; Ross, E.D. Sequence Features Governing Aggregation or Degradation of Prion-Like Proteins. *PLoS Genet.* **2018**, *14*, doi:10.1371/journal.pgen.1007517.
